# Advancing Chimeric Antigen Receptor T-Cell Therapy for Acute Myeloid Leukemia: Current Limitations and Emerging Strategies

**DOI:** 10.3390/ph17121629

**Published:** 2024-12-04

**Authors:** Daniela Damiani, Mario Tiribelli

**Affiliations:** 1 Division of Hematology and Stem Cell Transplantation, University Hospital, 33100 Udine, Italy; mario.tiribelli@uniud.it; 2 Department of Medicine (DMED), University of Udine, 33100 Udine, Italy

**Keywords:** CAR T-cell therapy, acute myeloid leukemia, toxicity, outcome

## Abstract

Chimeric antigen receptor (CAR) T-cell therapy represents one of the most impressive advances in anticancer therapy of the last decade. While CAR T-cells are gaining ground in various B cell malignancies, their use in acute myeloid leukemia (AML) remains limited, and no CAR-T product has yet received approval for AML. The main limitation of CAR-T therapy in AML is the lack of specific antigens that are expressed in leukemic cells but not in their healthy counterparts, such as hematopoietic stem cells (HSCs), as their targeting would result in an on-target/off-tumor toxicity. Moreover, the heterogeneity of AML and the tendency of blasts to modify surface antigens’ expression in the course of the disease make identification of suitable targets even more challenging. Lastly, AML’s immunosuppressive microenvironment dampens CAR-T therapeutic activities. In this review, we focus on the actual pitfalls of CAR T-cell therapy in AML, and we discuss promising approaches to overcome them.

## 1. Introduction

Acute myeloid leukemia (AML) is a rare malignancy characterized by multiple alterations of bone marrow (BM) precursors arising from genetic mutations that lead to clonal proliferation and acquisition of a neoplastic phenotype [1,2]. The SEER-reported age-adjusted incidence of AML has gradually risen over the last decades to 4 per 100,000 per year [3], in part as a consequence of the increased survival after cancer therapies [4]. Until a few years ago, the conventional chemotherapy regimen, established back in the 1970s and based on anthracycline in combination with cytarabine, resulted in a modest increase in long-term survival [5,6], justifying the extensive use of allogeneic hematopoietic cell transplantation as the best curative option in intermediate- and high-risk patients [7,8]. Only in recent years have advances in the knowledge of AML pathogenesis, refinements in risk stratification, and the identification of molecular markers as potential targets for new therapies led to an initial, though still unsatisfactory, increase in the survival rate [9,10]. Besides the intrinsic or acquired resistance to chemotherapy of leukemic cells, evidence of a permissive BM microenvironment protecting leukemia stem cells (LSCs) and favoring disease recurrence has highlighted the potential role of therapies harnessing the immune response to reverse the immunosuppressive microenvironment [11]. In this way, chimeric antigen receptor (CAR) T-cells represent an appealing approach.

CAR T-cell therapy is based on ex vivo T lymphocytes engineered and expanded to express CARs that, after infusion, recognize and kill tumor cells in an MHC-independent manner [12]. The first generation of CARs, consisting of the single-chain variable fragment (scFv) of an antibody linked to the intracellular CD3ζ signaling domain through a transmembrane domain (TMD), showed insufficient in vivo activation, expansion, and persistence [13]. Therefore, in the second-generation CARs, the signaling domain of co-stimulatory receptors, such as 4-1BB (CD137) and CD28, was added to the intracellular domain, with improved clinical results [14]. Currently, the FDA/EMA-approved CAR T-cell therapies are based on this construct.

However, in contrast to the impressive results in lymphoid diseases (relapsed/refractory ALL and B cell lymphomas) [11,15,16], the safety and the efficacy of CAR T-cells in AML, although encouraging, are still limited [9,17,18], underlining the different disease biology and the need for further refinements.

In this paper, we summarize the limitations to the use of CAR T-cells for AML treatment and discuss the possible strategies to increase their efficacy, including further modification of the CAR construct, the modulation of CAR T-cell persistence to limit off-target toxicity, increased recognition of leukemia-specific antigens, and methods to break immune tolerance in the AML BM.

Published data on CAR T-cells in AML are scarce and limited to heavily treated patients or refractory diseases. A summary of the available clinical data is shown in Table 1.

Evaluable data are mostly the result of Phase 1/1a clinical trials or case reports including very few patients. The most common target antigens are CD33, CD123, and CLL-1 (C-type lectin-like molecule-1), all usually expressed in more than 90% of leukemic cells, irrespective of French–American–British (FAB) classes, and on leukemic stem cells. However, there is great variability in study design, CAR-T construct, cell source and manufacturing, and lymphodepletion regimen. T-cell subtypes in apheresis were mostly not evaluated, and the biologic diversity of AMLs (such as cytotype, caryotypic, or molecular abnormalities) are not considered, so a comparison among trials and an evaluation of the actual CAR-T efficacy is very difficult. Furthermore, in AML, CAR T-cells have sometimes been used as “bridge therapy” to allogeneic SCT, further preventing an assessment of their efficacy in eradicating leukemia.

Compared to lymphoid neoplasms, in AMLs, CAR T-cells showed a greater frequency of prolonged and life-threatening myeloablation. This is first due to the disease itself, but it could be also affected by the CAR T-cell construct. Although a direct comparison is lacking, in lymphoid malignancies, the CD28 costimulatory domain seems to be associated with a shorter duration of CAR-T-induced myeloablation due to shorter CAR T-cell persistence [29]. On the other hand, accumulating evidence from the use of CD19-CAR T-cells in B cell leukemia suggests that durable clinical response and relapse risk depend on CAR T-cell persistence [30]. The ideal “persistence time” able to limit toxicity and maximize efficacy is far to be established. The “third generation” CAR T-cells was developed for the purpose of enhancing their efficacy by including two complementary costimulatory domains, usually CD28 and 41BB, along with the intracellular CD3ζ signaling domain [31]. In AML, this could be advantageous in low-burden diseases or in low antigen density, where the activation and persistence signals by tumor cells are weak and may benefit from additional co-stimulation. CD28 would ensure rapid expansion of T-cells and tumor cell clearance, while CD137 would provide longer persistence of CAR T-cells in the host [14,29]. Wuhan et al. performed a phase I trial on the safety and efficacy of anti-CD123 third-generation CAR T-cells in relapsed/refractory adult AML (NCT04014881). Recruiting is closed, but the results are not yet available. However, preliminary data from their use in lymphoid malignancies (NCT01853631, NCT02132624, NCT 03676504) did not prove a clear superiority of third-generation CAR T-cells compared to the second one, justifying the development of further CAR-T constructs (next-generation CARs) planned to increase their efficacy and reduce treatment-related adverse events.

## 2. Limitations and Challenges of CAR T-Cell Therapy in AML

The first barrier to translating the success of CAR T-cell therapy to AML is the lack of suitable target antigens. The ideal target for immunotherapy (antibody- or cell-mediated) should (1) identify only neoplastic cells and spare healthy tissues, in particular the normal hematopoietic compartment; (2) be expressed in the majority of AML cases; (3) be highly expressed in LSCs and AML progenitor cells, the subpopulation of cells with the greatest self-renewal and drug resistance capacity; and (4) be involved in leukemogenesis so that downregulation of the target antigen would result in a survival disadvantage for leukemic cells.

However, compared to most lymphoid malignancies, AML is genetically more heterogenous, as driver oncogenic mutations significantly vary among patients, and patients may harbor multiple leukemic clones [9,32], making a therapy targeting a single antigen unlikely to eradicate all of the malignant clones. Sometimes, the AML mutation profile can be linked to the expression of specific antigens, such as CD123 and NPM1 mutation [33], and sometimes antigen expression is associated with the maturation stage of leukemic cells. To this group belong the targets currently used in the early clinical trials (CD33, CD123, FLT3). Unfortunately, most of them are also expressed on healthy hematopoietic stem cells, explaining the prolonged myeloablation that complicates CAR T-cell therapy in AML. Leukemia-associated antigens, such as WT1 and PRAME, are overexpressed on leukemic cells compared to normal cells, and they are usually not lineage-specific, so they are less probably expressed in a normal hematopoietic compartment and thus less likely to cause severe and prolonged aplasia. Early phase trials on WT1 and PRAME are ongoing in different immunotherapy settings, but not in CAR T-cells [34,35,36]. Leukemia-specific neo-antigens are intracellular and presented in the context of HLA molecules. They are aberrant proteins resulting from mutated genes expressed exclusively in neoplastic clones. A spontaneous immune response mediated by CD4+ or CD8+ T-cells has been reported for many, but not all, of them [37,38,39,40,41,42]. Whether this effect is leukemia-specific (i.e., against HLA antigens) or not is far to be clarified [43]. To date, no CAR T-cell trials against the known leukemia-specific antigens are ongoing.

The partial or complete loss of the CAR target on the cell surface is a further challenge for CAR T-cell therapy. The absence of a target antigen may be explained by the emergence of pre-existing negative leukemic clones under therapeutic pressure or downregulation of the target, which is used by leukemic cells to evade the immune system and to resist death [44]. Another limit to CAR-T efficacy in AML is the low expression of target antigens, resulting in defective activation and expansion of CAR T-cells, a phenomenon that can only be partly overcome by increasing the number of costimulatory domains in the CAR construct. In the lymphoid setting, a low antigen expression correlated with the inability of CAR-T to eliminate neoplastic cells, as proven by the coexistence of persistent CAR T-cells and leukemic cells in BM [45]. Walker et al. demonstrated that a high target antigen density (>10,000 molecules/cell) was needed to fully activate CAR T-cells’ killing activity [46]. Antigen loss and antigen-low escape are likely to emerge as an even greater barrier to the success of CAR-T therapies in solid tumors and in AML, diseases both characterized by greater heterogeneity in target antigen expression.

Besides the need for increased CAR-T “specificity”, the other hurdle in employing autologous CAR T-cells is the so-called “off-tumor toxicity”, namely cytokine release syndrome (CRS) and immune effector cell-associated neurotoxicity syndrome (ICANS). CRS is the most common adverse event across the trials of CAR T-cell therapy [47], and it is a systemic inflammatory response with variable clinical manifestations ranging from mild symptoms to severe multiorgan failure. Moreover, CRS can be considered an “initiating event” or a cofactor for ICANS that in general occurs after CRS, although concurrent presentation of CRS and ICANS can occur. An exhaustive description of the pathogenesis, clinical aspects, and management of CRS has been provided by Neelapu et al. [48] and Titov et al. [47].

Last but not least, the manufacturing of a CAR-T product for AML therapy may also present a challenge, especially for refractory AML; in such patients, a reduction in the manufacturing time (typically 3–5 weeks “vein to vein”) is critical to minimize the toxicity of the bridging therapy needed to control the disease before cell therapy.

## 3. Overcoming Limitations and Managing Toxicities

### 3.1. Target Antigen Identification

**Figure 1:**
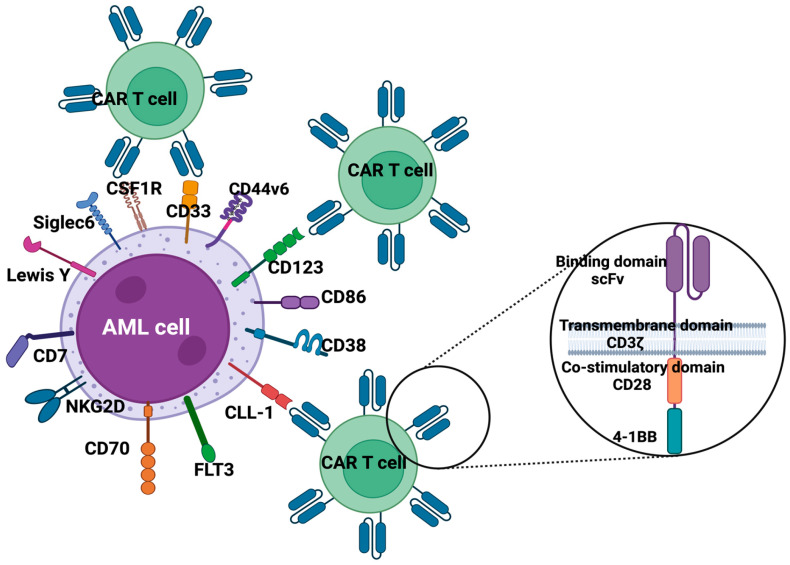
Schematic representation of known and potential targets on myeloid leukemia cells.

Identifying leukemia-specific neo-antigens represents the first step to improving the efficacy of CAR T-cells for AML therapy by avoiding on-target/off-tumor toxicity. Neo-antigens are peptides derived from aberrant cancer-specific proteins through a complex multi-step intracellular process through which they are loaded onto HLA molecules on the cell surface to be presented to T-cells [49]. Neo-antigen can potentially be generated by any single amino acid mutation, fusion proteins, or cancer-specific isoforms, but not all aberrant proteins yield neo-antigens. Moreover, as the neo-antigen is part of a protein crucial for maintaining the malignant phenotype, tumor escape through the loss of the target antigen is less probable. However, there are many limitations to the therapeutic use of neo-antigens in hematologic malignancies. First, it must be remembered that in AML, the frequency of mutations, and thus of potential neo-antigens, is forty time less common compared to some solid tumors, such as lung cancer and melanoma, that may carry hundreds of mutations in each individual [50,51]. Second, targeting leukemia-specific neo-antigens may require “individual” manufacturing of the therapeutic tool. Finally, alternative ways to escape the immune system are always possible, including down-regulation of HLA molecules [52,53,54] or altered proteasomal processing of the epitope [55]. Despite this, technological progress has permitted the identification of some potential targets, and, at present, many molecules are under preclinical and early clinical investigation as candidates for CAR T-cell therapy. A summary of CAR-T potential targets is shown in Figure 1.

None of them fulfill all of the requirements for an ideal antigen, but potential leukemia-specific antigens are those derived from alternative mRNA splicing, such as CD44v6, or molecules that are upregulated or subcellularly re-located upon cellular stress (PR1 peptide, GRP78) or chemical induction (CD38, FRβ) [56]. Gottschlich et al. screened AML samples through single-cell RNA sequencing (sc-RNA-seq) and identified CSF1R and CD86 as potential targets; they tested CAR T-cells in AML cell lines and in patient-derived xenograft models, with promising results [57]. Hebbar et al. tested in vitro and in vivo glucose-regulated protein 78 (GP78) demonstrating anti-AML efficacy and protection of HSCs [58]. Trad et al. proved, both in vitro and in xenografts models, the effectiveness of CAR-T against interlukin-1 receptor accessory protein (IL1-RAP). They proposed IL1-RAP as an attractive target for its expression on LSCs and blasts but not on HSCs [59]. John et al. developed CAR T-cells against leukocyte immunoglobulin-like receptor B4 (LILRB4), which is highly expressed on monocytic AML cells and is known to cause T-cell suppression and AML tissue infiltration. They demonstrated high anti-AML activity and, in a humanized hematopoietic-reconstituted mouse model, absence of toxicity to normal hematopoietic cells [60].

Innovation in high-throughput genomic and transcriptomic sequencing technologies has significantly facilitated the identification of protein-coding mutations and fusions producing potential neo-antigens, despite reliable in silico methods to prove their immunogenicity are still lacking. The Cancer Genome Atlas Research Network conducted an extensive analysis of the mutational landscape of AML, identifying recurrent mutations involved in the leukemogenesis pathway [61]. About 30–35% of AML cases harbor mutations in the nucleophosmin (*NPM1*) gene. Most mutations comprise the insertion of four nucleotides in exon12 that produce a novel C terminal amino acid sequence [62]. From a functional point of view, this leads to a nuclear export signal with localization of the mutated protein in the cytoplasm [37]. Eighty-five percent of patients with mutated NPM1 (NPM1^mut^) share this type of mutation that produces an identical aminoacidic sequence. Moreover, mutation is stable across the disease course and is considered a driving mutation, making it an excellent immunotherapy target [38]. Van der Lee et al. identified CD8+ T-cell clones from healthy donors specific for the NPM1^mut^ HLA-A*02:01 epitopes and demonstrated that epitopes presented with HLA molecules on leukemic cells are immunogenic (i.e., neo-antigens). Xie et al. developed a CAR construct specific for the NPM1^mut^-HLA-A2 complex. Both in vitro and in mouse models, these CAR T-cells exhibit great cytotoxicity against NPM1^mut^ cell lines and primary AML blast cells, but not in NPM^wt^ leukemic cells or in HLA-A2-negative cells [63]. The PML-RARA protein, resulting from the fusion of the retinoic acid receptor alpha (RARA) gene on chromosome 17 and the promyelocytic leukemia (PML) gene on chromosome 15, has also been proposed as a potential neo-antigen for cell therapy. Gambacorti-Passerini et al. demonstrated the proliferation of a CD4+ T-cell clone in response to PML-RARA fusion protein in association with HLA-DR*11 exposure [41]. However, the PML-RARA/HLA-DR-specific T-cell activation was not confirmed in a successive study [64], so the inclusion of PML-RARA among potential neo-antigens is still pending. Graf et al. proposed as a neo-antigen an epitope generated from FLT3 internal tandem duplication (ITD), a mutation observed in about 30% of AML patients [40]. They tested the immunogenicity of YVDFREYEYY peptide restricted by HLA-A*0101 in an autologous setting, demonstrating a CD8+ T-cell response. Isocitrate dehydrogenase type 1 (IDH1) mutation has been observed in 6–16% of adult patients with AML and is frequently associated with NPM1, DNTM3A, and FLT3-ITD mutations, an association that leads to inferior outcome [65]. Schumacher et al. showed that the IDH1 R132H mutation contains immunogenic epitopes suitable for immunotherapy. Peptides including the mutated region were presented with MHC class II molecules and elicited a mutated specific CD4+ (TH1) immune response in mice models with IDH1 mutated gliomas, making it a possible neo-antigen for IDH1 mutated AMLs [39]. The t(6;9)(p23q34) translocation, generating the DEK::NUP214 fusion protein, is found in about 1% of adult AML patients, often in association with FLT3-ITD mutation, and it defines an extremely aggressive disease [66]. Non-fusion DEK proteins support stem cell maintenance and promote engraftment after allogenic transplantation. DEK fusion proteins cause the dysregulation of myelopoiesis and promote leukemogenesis [66]. Makita et al. demonstrated that DEK::NUP214 fusion peptide, presented in association with HLA class II, induced IFN-γ release by CD4+ T-cells, and it can be a target for immune therapy [42]. Dysregulated splicing resulting in a protein isoform distinguishable from the wild-type form may represent a source of potential neo-antigens. A fusion between *CBFB* and *MYH11* genes, resulting from inv (16) and t(16;16), occurs in 7% of adult and in 12% of pediatric AML [67,68]. This fusion is an essential leukemia-initiating event occurring in the AML founding clone, and it is stable across the disease course and persists at relapse [69,70,71,72]. Biernacki et al. demonstrated that the peptide REEMVEHEL is present on HLA-B*40:01^+^, activating highly specific CD8+ T-cell clones able to recognize and kill AML cells in vitro and in vivo, making it a potential target for CAR T-cell therapy [73]. Adamia et al. demonstrated a variant of Notch homolog protein 2 (Notch2-Va) and a variant of FLT3 (FLT3-Va) on AML blast cells [74], representing potential disease markers and targets for new therapies, including CAR T-cell therapy. Finally, some mutations involving oncogenes or tumor suppressor genes may generate neo-antigens shared among patients with different cancers, including some hematologic neoplasms. For instance, KRAS and NRAS mutations are found in about 5–26% of hematologic diseases, and RAS mutation has been observed in 16% of AML and 5% of MDS patients [75]. In solid tumors, a CD4+ T-cell response restricted for an HLA-DRB1*0801-restricted epitope from the KRAS G2D mutation and a CD8+ T-cell response for HLA-A*11:01 and the G12V peptide have been reported [76]. This information has already been translated into clinical trials (NCT03190941 and NCT3745326), but it may also find application in AML, where the codon G12 mutation of RAS results in an amino acid sequence identical to KRAS G12D and G12V. The TP53 protein is involved in many physiological roles, including DNA repair, cell cycle arrest, apoptosis, glucose and lipid metabolism, control of stemness, lineage commitment, and differentiation, triggered in response to various cellular stresses [77]. TP53 mutations are present in 12% of AML and 6% of MDS patients. While TP53 mutations may be heterogeneous, there are some mutational hotspots (R175, R245, R248, R273, R282) that are shared across cancers, including AML [78]. Malekzadeh et al. identified an HLA-A*02:01 restricted epitope from the R175H mutation able to generate a T-cell response [79]. Moreover, immunogenic HLA-restricted epitopes have been identified that also form a sample of patients with solid tumors [80]. Because the codon distribution of TP53 mutations is not specific for cancer tissues, identified neo-antigens should also be used in AML.

A novel strategy to obtain leukemia-specific antigens has been proposed by Kim et al. They proposed to create an “artificial” leukemia-specific antigen by editing out CD33 from normal HSCs, thereby generating a hematopoiesis resistant to anti-CD33 therapy and enabling specific targeting of AML with CD33 CAR T-cells [81]. In mouse models, CD33 knockout does not affect the differentiation ability, maintenance, or engraftment capacity of normal stem cells, and CD33-CAR T-cells cause a rapid clearance of leukemia cells in mice engrafted with CD33 KO HSCs. A phase I clinical trial investigating the efficacy of allogeneic CD33 CAR T-cells in patients with R/R AML previously engrafted with CD33KO HSCs is ongoing; no data are available at present (NCT05945849). A similar approach may be used with the CD123 molecule. However, CD123 is involved in hematopoietic development, and its complete deletion could negatively impact the HSC compartment [82]. Alternative approaches could be the removal of just the CD123 epitope recognized by CAR T-cells or permitting its expression at low levels (below the CAR-T activation threshold), thus preserving normal hematopoiesis.

### 3.2. Overcoming Heterogeneity: Dual-Targeting and Logic Gating

The combination of CARs against different AML-associated targets may overcome the lack of leukemia-specific antigens. Targeting multiple antigens (two or more) can be achieved through sequential infusion of two different CAR T-cells or by engineering multi-specific T-cells in which the CAR construct has been modified to simultaneously bind two antigens. The administration of multiple validated CAR T-cells, at same time or sequentially, is attractive for mixing and matching different cell products. Several dual-targeting CAR T-cells for B cell lymphomas, ALL, and multiple myeloma (MM) have reached preclinical and early clinical phases and have induced some long-term responses, improving OS [83,84,85,86] with an acceptable safety profile [87] but without a significant improvement compared to single CAR T-cell administration. The co-infusion of different CAR T-cell products requires further investigation to adjust the doses and the time of infusion and to study the synergy or dominance of each product.

**Figure 2:**
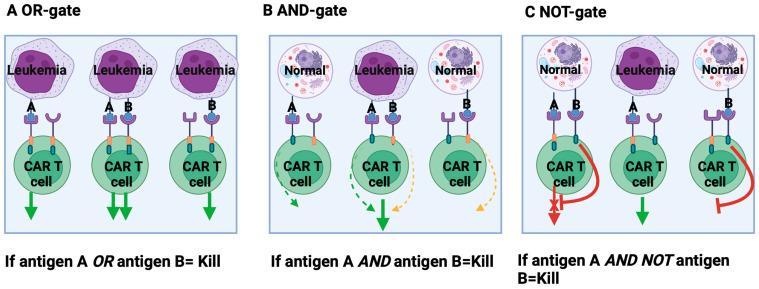
Principles of logic-gating to improve target recognition and limit “off leukemia” toxicity. (**A**) OR-gate, where CARs target distinct leukemia antigens A and B and CAR T-cells are activated through recognition of one or both of them. (**B**) AND-gate, where CAR T-cells are fully activated when CAR T-cells engage both A and B antigens, expressed on leukemia, but not on normal cells. (**C**) NOT-gate, which involves T-cell co-expression of a CAR specific for antigen A and an inhibitory CAR specific for antigen B. T-cells are fully activated when CAR engages with antigen A on tumor cells. Engagement with antigen B expressed on normal cells inhibits CAR T-cells. Green continuous arrow: activation; red continuous arrow: inhibition; dotted green arrow: weak activation; yellow dotted arrow: alternative weak activation.

These challenges supported the rationale of engineering multi-target CAR T-cells, which are also potentially useful in cases with low expression of single targets. The first method for concomitant antigen targeting, in which either one of a pair of antigens works as the target, is defined as an OR-gate in Boolean terminology. In Figure 2 is reported a schematic representation of the different “gating” methods useful for improving target recognition and limiting toxicity.

T-cells can express two distinct, fully functional CARs targeting different antigens (Dual-CAR) or a single bivalent CAR, tandem binding domains (Tan-CAR). At present, there are no data regarding the superiority of one compared to the other. Dual-CAR seems efficient in avoiding antigen escape, but it may present reduced signal activation due to the competition for downstream signaling molecules. In Tan-CAR, the CAR structure may negatively affect antigen binding. In AML, bi-cistronic CAR T, in which a single vector was transduced to produce Bi-CAR-T targeting CD123 and CD33, showed significant anti-tumor activity in cell lines and in vivo [88]. Liu et al. conducted a phase 1 study in R/R AML with Bi-CAR T-cells directed against CLL1 and CD33, obtaining the complete disappearance of blast cells in BM and peripheral blood [27]. Zhang et al. obtained a response in 10/11 pediatric patients with R/R AML and MDR negativity in 5 out of 10, without dose-limiting toxicities [89]. Atilla et al. observed higher anti-tumor activity in cells with low antigen expression in all tested dual combinations. Because the expression of antigens is variable, they chose to use different vectors to obtain variable molar ratios of the CAR constructs and different E:T ratios [90]. Li et al. explored the preclinical efficacy of FLT3/NKG2D CAR-T for FLT3 mutated AML. They observed the elimination of both FLT3 mutated and unmutated AML blasts because of the synergistic effect of the two targets [91]. Other studies investigated different combinations of CAR targets: CD13/TIM3, CD123/FRβ, IL3-zetakine/CD33, and CD123/NKG2D [92,93,94,95]. Despite their in vitro efficacy, in general, combinational strategies of CAR T-cells recognizing any of two targets increase T-cell activity and minimize the risk of antigen escape at the cost of the accumulating toxicities of each antigen. Combinatorial strategies restricting T-cell reactivity to cells expressing both of the targets reduced toxicity against cells expressing one target but required the expression of the two targets in all leukemic cells. [96]. Perna et al. proposed a new combinatory strategy in which targets with non-overlapping expression in normal tissues were paired to limit off-tumor toxicity. Integrating proteomics and transcriptomics analysis, they identify four possible pairs, three of which stained over 97% of AML cells and less than 5% of normal HSCs and T-cells [97]. To by-pass this limitation, other OR-gate constructs are under investigation.

In parallel, the CAR-T activation signal derives from the heterodimerization of a fully functional second-generation CAR construct and of a chimeric costimulatory domain with the same hinge region to prevent conflicts in downstream signals. In adapter-CAR-T, a leucine zipper adapter is inserted between the extracellular domain and the membrane domain to allow for flexibility in target binding, thus facilitating the engagement of multiple targets and providing adequate activation signals. Nixdorf et al. recently reported the effectiveness of such a technology using anti-CD33, anti-CD123, and anti-CLL1 adapter molecules to target AML in vitro and in murine models [98]. Finally, CAR-Bite-CAR T-cells combining a traditional CAR construct with a bispecific T-cell engager have been proposed to prevent antigen escape [99].

Another way to increase leukemic cell engagement from a set of non-specific targets is based on the Boolean AND-gate. In this model, a dual CAR consists of two separate CAR molecules with specificity to different antigens. The full activation of CAR T-cells depends on the combination of inputs from two antigens: the first provides only the activation signal (like first-generation CAR-T) and the second provides co-stimulation [100]. In preclinical mouse models, this system resulted in prolonged survival in AML treated with AND-gated CARs targeting CLL1 and CD33 or CLL1 and CD123 [90]. The major flaw is the possibility of leukemia escape favored by the heterogeneity of targets’ expression and the possibility that an antigen expressed on normal tissue might elicit an activation signal. So, a second generation of AND-gate CAR that uses sequential signals has been developed under the control of an inducible promoter activated only by signals mediated from a constitutively expressed synthetic Notch receptor (syn Notch). The binding of target antigen cleaves the Notch core region and releases a transcription factor from the cell membrane that migrates into the nucleus and drives the expression of an “effector” CAR. The syn Notch system has been applied to many cancer models, including leukemia [101,102,103]. In addition to more precise tumor targeting, the intermittent expression of a CAR mediated by syn Notch may prevent CAR-T exhaustion due to tonic activation produced by constitutively expressed CARs [103].

Better discrimination between tumors and healthy cells can be obtained by applying a NOT-gate, especially when leukemia/tumor antigens are expressed on normal tissues. This form of gating depends on an inhibitory CAR (iCAR), which prevents cell lysis if it binds a normal antigen. The major obstacle in developing this type of gating is the identification of an antigen with strong expression on leukemia/tumor cells. Richards et al. employed a NOT-gate strategy to kill CD93-positive AML cells, sparing the toxicities on endothelial cells [104].

### 3.3. Overcoming Antigen Loss/Antigen Low Expression Density

Targeting more than one antigen on leukemic cells may be also an effective strategy to counteract antigen loss after T-cell therapy. Tandem CAR-T has been developed to target HER-2 and IL13Rα2 in solid tumors [105], bispecific CD19/CD20 has been designed for B cell malignancies [106], and, more recently, a trivalent CAR T-cell construct has been developed for glioblastoma [107]. Pros and cons of these combinatorial approaches have been discussed above. In the case of low-density antigen expression, the efficacy of CAR T-cells might be increased by engineering CAR-T to respond to lower-density expression by enhancing ScFv for its target. However, whether this translates into higher CAR-T activity has to be proven [108,109,110]. An alternative approach, explored in vitro, may be pharmacologically induced target antigen expression. In preclinical studies, all-trans retinoic acid has been used to increase folate receptor β (FRβ) in AML [111], while bryostatin can increase CD22 expression B cell leukemia and lymphoma cells, and it seems to prevent the emergence of low CD22 variant subclones [110,112].

### 3.4. Enhancing CAR T-Cell Persistence

Exhaustion, which impairs killing activity and in vivo long-term persistence of CAR T-cells, partly explains the limited results in AML and in solid tumors. Exhaustion and dysfunction of CAR T-cells can be caused by high levels of ligand-independent tonic signals generated by the self-aggregation of CARs, which promote “continuous” CAR proliferation [113,114]. Other types of self-activating CAR T-cells have an exhausted phenotype, impaired in vitro proliferation, and upregulation of inhibitory molecules, such as PD-1, TIM-3, and LAG-3. Both self-activating CAR-Ts show good in vitro cytotoxicity but poor in vivo activity [113,115]. Self-aggregation could be prevented through modification of the structure of the CAR extracellular domain, scFv [116], or by changing the hinge and TMD [117]. In addition, a mitigation of tonic Ag-independent signals and the restoration of the cytolytic ability can be attained through modification of the spacer between scFv and TMD [118]. Alternative potential ways to stop tonic CAR signals are to suspend CAR expression when it is unnecessary [119] and modification of downstream signals [120]. Chen et al. demonstrated a correlation between the expression of the NR4A transcription factor and the expression of inhibitory molecules. Moreover, in murine models, NR4A knockout CAR T-cells decreased the proportion of exhausted CAR T-cells and improved the prognosis [120].

### 3.5. Counteracting the Immunosuppressive AML Microenvironment

Over the past ten years, it has become evident that a permissive BM microenvironment plays a crucial role in the development of acute myeloid leukemia and, in response to chemotherapy, facilitating leukemia cells’ escape from immune control and supporting the persistence of leukemia stem cells responsible for disease recurrence [121]. The more recent studies employing CAR T-cells suggest that the immunosuppressive AML microenvironment can also negatively impact the antitumor activity of CAR T-cells. Suppressive immune cells, including T regulatory cells, myeloid-derived suppressor cells, and M2 polarized macrophages, are involved in effector T-cell inhibition. Soluble environmental factors, including anti-inflammatory cytokines, chemokines, metabolic changes, and overexpression of checkpoint inhibitors, contribute to T-cell exhaustion, reducing T-cell activity, persistence, and localization. A detailed description of microenvironmental factors able to reduce CAR T-cell efficacy can be found in the paper by Epperly et al. [122].

AML blast cells express many inhibitory signals, such as PD1-L1, B7-H3, and galectin 9. The interaction with the respective ligands inhibits T-cell activation. In AML, PD1 knockdown in CLL1 CAR T-cells showed potent anti-leukemia activity in vitro and limited side effects [123]. Ma et al. treated two AML patients relapsed after transplant with these CAR T-cells; both patients achieved CRi lasting 8 and 3 months, respectively [124]. The possibility of generating antigen-specific iCAR, which inhibits checkpoint molecules, such as PD-1 and CTLA4, seems to be a promising approach [125]. The use of “armored” CAR T-cells makes AML cells more susceptible to CAR-T-mediated lysis, but it also acts on CAR T-cells, ensuring long-term activation and persistence. In the NCT03258047 clinical trial, CAR was obtained by fusing the extracellular domain of PD-1 to the intracellular activating domain of CD28, and the interaction with its ligand PD1-L1 generated an activation rather than an inhibitory signal [126]. The study involved 17 patients with B cell lymphoma. CR and ORR responses were observed in 41% and 58%, respectively, with an apparent superiority compared to traditional CAR T-cells. No trials using “armored” CAR T-cells are ongoing in AML. An alternative strategy is to disrupt PD-1 signaling by programming CAR T-cells to secrete PD-1 Fc fusion protein, which binds PD1-L1 and prevents T-cell immunosuppression; it is under investigation in MM and B cell lymphoma (NCT04162119 and NCT04163302), but results are not yet available. CAR-T-cell-secreting IL12 and IL18, defined as either “Armored CAR-T” or “T-cell redirected for universal cytokine-mediated killing” (TRUCKs), have been recently designed to stimulate inflammation and inhibit immunosuppressive cells. They have demonstrated enhanced anti-tumor efficacy in preclinical models [127]. IL12 also counteracted immunosuppression of Tregs and myeloid-derived suppressor cells [127]. Avanzi et al. designed CAR T-cells secreting IL18, which increased the production of gamma-IFN and modulated the microenvironment, thus enhancing the endogenous immune response [128]. Currently, six clinical trials are ongoing to evaluate the TRUCK approach, all in lymphoid malignancies and in MM. Preliminary results showed a high CR rate in lymphoma and 100% CR in MM [129,130]. Beyond impairing CAR T-cell function, the immunosuppressive microenvironment of AML can negatively affect CAR T-cells’ persistence. Although not planned for AML, many strategies have been shown to improve CAR T-cell expansion and persistence, including the expression of IL15 or IL18 and the addition of exogenous IL7, IL15, and IL21 during ex vivo CAR T-cell expansion [131,132,133,134]. Kuhn et al. developed CAR T-cells expressing CD40L able to engage CD40+ tumor cells, resulting in direct cytotoxicity or antigen-presenting cells (APCs) [135]. Lai et al. engineered CAR T-cells to secret the FLT3 ligand, which expanded dendritic cells and activated endogenous antitumor T-cells [136].

### 3.6. Overcoming Toxicity

CAR-T-cell-mediated toxicities can be severe and life-threatening. Many promising strategies to control CAR T-cells’ persistence after infusion include mRNA electroporation, antibody-mediated depletion, suicide switches, and drug-inducible switches. The mRNA-electroporated CAR T-cells have short lifespans but a low risk of persistent toxicity. In the NCT02623583 trial, multiple infusions of biodegradable (electroporated) CD123 CAR T-cells in R/R AML demonstrated their biologic effect (fever and CRS), but they showed a lack of anti-leukemic activity due to their short lifetime [137]. An antibody-mediated depletion of CAR T-cells can be obtained by incorporating a truncated CD20 molecule into a CAR cell that can be recognized by an anti-CD20 antibody, i.e., rituximab. [138]. In addition to CD20, a truncated version of epidermal growth factor receptor (EGFR), recognized by the commercial anti-EGFR drug cetuximab, has been successfully used in preclinical studies [139]. Two trials investigating EGFR–cetuximab suicide CAR-T are ongoing (NCT03314670, NCT02159495). The incorporation of inducible caspase9 (icasp9) represents an alternative approach. Administration of the lipid-permeable tacrolimus analogue rimiducid induces caspase dimerization and triggers apoptosis [139]. The rescue strategies described above result in irreversible loss of CAR T-cells. Leung et al. developed a method leading to transient CAR-T control. They produced dimerizing regulated immunoreceptor complex (DARIC) CARs in which the antigen binding and the intracellular subunit of BAR are split. Under rapamycin exposure, they dimerize and trigger activation [140]. In xenograft models with a CD33 target, DARIC CAR T-cells provided excellent disease control. A clinical trial (NCT0510512) is ongoing with DARC-CARS.

### 3.7. Combination Therapies

The still unsatisfactory results of CAR T-cells therapy in AML, their toxicity, and the complexity of the BM microenvironment in AML are the rationale for combination therapies, which might directly or indirectly enhance CAR T-cell activity through a synergistic attack on leukemia cells by overcoming the resistance mechanisms. Several studies have tested the efficacy of the combination of CAR T-cells and target therapies in hematologic malignancies. El Khawanky et al. employed a pretreatment with azacytidine (AZA) to increase CD123 expression on AML cells but not in normal HSCs and precursors in a murine model, thus enhancing the activity of CD123 CAR T-cells [141]. Xu et al. demonstrated that the co-culture of AZA and leukemic cells increased the expression of OX40L (CD252) on AML blasts and enhanced CAR T-cells’ killing through the OX40/OX40l costimulatory pathway [142]. Wang et al. observed that exposure of CAR T-cells to low-dose decitabine favored both a higher expression of genes related to memory cells and a lower expression of genes related to exhaustion, resulting in improved cytotoxicity and reduced depletion after antigen exposure [143]. Similarly, pretreatment with histone deacetylase inhibitors enhanced NKG2D ligands on AML cells and increased the anti-leukemia activity of NKG2D-CAR T-cells [144]. Jetani et al. observed the superior efficacy of FLT3-CAR T-cells in combination with the FLT3 inhibitor crenolanib [145]. However, they also reported a depletion of HSCs, suggesting the use of this combination as a bridge to transplantation. Li et al. found that the FLT3 inhibitor gilteritinib induces the expression of NKG2DL on leukemia cells and constructed an AND-gate NKG2d/FLT3 dual CAR to target FLT3-mutated cells, which eradicated leukemia in a mouse model [91]. Many other molecular targeted drugs, including tyrosine kinase inhibitors, Bruton kinase inhibitors, PI3k inhibitors, BCL2 inhibitors, JAK inhibitors, and checkpoint inhibitors, demonstrated in vitro and in vivo synergistic activity with CAR T-cells in various settings, and they can also potentially be used in AML. An exhaustive review of currently available data on combination therapies has been recently published by Huang et al. [146].

### 3.8. Optimizing Manufacturing: Moving to Universal CAR T-Cells

At present, approved and under investigation conventional CAR-T products are manufactured from autologous T-cells derived from the intended recipient patients. However, this personalized production has limitations that risk hampering dissemination and equal access to this revolutionary therapeutic tool. The first problem is the costs of production. An evaluation of costs in six European countries published by Heine et al. in 2021 estimated the budget impact of CAR T-cell therapies in the next decade as high as EUR 28.9 billion. Considering possible new indications and the consequent increase in potentially eligible populations, the authors raised serious concerns regarding the financial sustainability and highlighted the need for changes in the allocation of healthcare budgets [147]. The second problem is the duration of the manufacturing process. The current “vein to vein” time of 2 weeks may affect eligibility, especially in patients with rapidly progressing disease, such as R/R AML [148]. The third question is the quality of the apheresis product, which is largely impacted by previous therapies and by disease-dependent T-cell dysfunctions, which can negatively influence CAR T-cell expansion and their ability to kill leukemic cells. The availability of “universal”, “off the shelf” allogeneic Universal CAR-T (UCAR-T) cells may increase the quality and accessibility of CAR T-cell products. UCAR T-cells have many potential advantages, including the immediate availability of cryopreserved batches and shorter duration and lower costs of production. The main limitations are, at present, inferior amplification and reduced persistence in vivo [149]. Gene editing technologies are the basis of strategies to reduce CAR-T-induced graft versus host disease and to make allogeneic cells invisible to the host immune system. At present, five trials investigating the feasibility, safety, efficacy, and persistence of U-CAR-T in R/R AML are recruiting (NCT05995041, NCT04230265, NCT05995028, NCT03190278, and NCT05949125).

## 4. Conclusions

Several clinical and preclinical studies have demonstrated the potential of CAR T-cells therapy for AML. The disappointing results of early clinical trials might, at least in part, depend to their use in heavily pre-treated R/R disease and cannot automatically be extrapolated to other settings. Despite the significant improvement in the knowledge of the mechanism of action and in the management of side-effects deriving from CAR T-cells use in lymphoid malignancies, many challenges must be overcome in AML due to the heterogeneity of the disease and the heavy impact of the AML immunosuppressive microenvironment on cellular therapies. So, new strategies specific for AML should be developed. Future research agendas should be focused on 1. enhancing/individualizing leukemia antigen recognition by considering multitarget CAR T-cells or including combination therapies able to increase leukemia antigen expression, and 2. the identification of the best allocation of CAR-T therapy along with the therapy program, i.e., as a bridge to allogeneic stem cell transplantation, after allogeneic stem cell transplantation to control minimal residual disease, or as alternative to allogeneic stem cell transplantation, possibly using allogeneic CAR T-cells. Lastly, the identification of biomarkers predicting outcomes and side effects is mandatory to maximize the full potential of CAR T-cells.

## Figures and Tables

**Table 1:** Summary of early clinical data on CAR T-cells in AML.

Target	Costimulatory Domain	Source	LD Regimen	Patients/Disease Status	Post CAR-T alloSCT	Efficacy	Safety	Ref.
CD33	41BB	auto	Flu 30 mg/m^2^ and Cy 500 mg/m^2^ × 3 days	3/RR	No	3/3 DP	CRS 66% (G3: none) ICANS: 33% (G3–4: none)	[19]
CD123	41BB	allo	Flu 30 mg/m^2^, Cy 750 mg/m^2^, and Ale 12 mg/m^2^ × 4 days	17/RR	//	1/9 CR; 1: 90% BM blast cell reduction	CRS 100% (≥G3 24%; G5: 12%) ICANS 6% (G3: 6%)	[20]
CD123	CD28	allo	Flu and Cy × 3 days *	14/RR	//	2/14 CRi; 1 MRD-negative	CRS 86% (G2 7%; G3 7%) ICANS: 7%	[21]
CD123	41BB	allo	RIC regimen	1/RR	//	CRi	CRS (G4)	[22]
CD123	CD28	auto	Flu and Cy	5/RR	No	1CR; 1 blast cell reduction; 1MLFS	CRS 100% ICANS: none	[23]
CD123	CD28	auto	Flu 30 mg/m^2^ and Cy 300 mg/m^2^ × 3 days	7/RR	1/7	1 CRi; 2MLFS	CRS 83% (G3/4: 0%) ICANS none	[24]
CLL1	CD28/CD27	auto	Flu 30 mg/m^2^ and Cy 900 mg/m^2^ × 4 days	4/RR	1/4	3CR	CRS 75%; (G3/4: none) ICANS: G1/2 25%	[25]
CLL1	41BB	auto	Flu 30 mg/m^2^ and Cy 900 mg/m^2^ × 4 days	3/RR	2/3	2CR	CRS 75%; (G3/4: none) ICANS: G1/2 25%	[25]
CLL1	41BB	auto	Flu 30 mg/m^2^ and Cy 500 mg/m^2^ × 3 days	10/RR	6/10	70% CRi (in 9/10 SCT by day 34)	CRS 100%; (G3 60%) ICANS none	[26]
CD33/ CLL1	Not defined	auto	Flu 30 mg/m^2^ and Cy 300 mg/m^2^ × 3 days	9/RR	No	7/9 MRD negative	CRS 89% (G3 22%) ICANS 44% (G3 33%)	[27]
NKG2D	Endogenous DAP10	auto	None	16/RR	2/16	3/12 CR	CRS 94% (G3 25%, G4 13%) ICANS none	[28]

* Doses not listed; LD: lymphodepletion; auto: autologous; allo: allogeneic; Flu: Fludarabine; Cy: cyclophosphamide; Ale: alemtuxumab; CR: complete remission; CRi: complete remission with incomplete hematologic recovery; DP: disease progression; BM: bone marrow; MLFS: morphological leukemia-free status; MRD: minimal residual disease; CRS: cytokine release syndrome; ICANS: immune-cell-associated neurotoxicity syndrome; SCT: stem cell transplantation.
